# Is “Just-in-Case” catheterisation inappropriate or system-driven? A human factors perspective – Authors’ reply

**DOI:** 10.1016/j.lanepe.2026.101817

**Published:** 2026-08-11

**Authors:** Bertrand Drugeon, Olivier Mimoz

**Affiliations:** a CHU de Poitiers, Service des Urgences Adultes – SAS 86 - Centre 15, Poitiers, France; b INSERM, U1070, Pharmacologie des Agents anti-Infectieux et Resistance, Poitiers, France; c Alliance for Vascular Access Teaching and Research, Griffith University, Nathan, Australia; d Université de Poitiers, Faculté de médecine et de pharmacie, Poitiers, France

We thank the authors for their interest in our study and for their thoughtful and constructive perspective.

We agree that anticipatory peripheral intravenous catheter (PIVC) insertion may, in some contexts, represent an adaptive response to resource constraints and time pressure in overcrowded emergency departments (EDs), as highlighted through a human factors lens. From a human factors perspective, such practices may represent a “work-as-done” adaptation to system constraints; however, our data suggest that this adaptation is not always aligned with optimal patient care. This interpretation should therefore be approached with caution. While such practices may preserve flexibility, they do not ensure clinical appropriateness. Our findings suggest that anticipatory PIVC insertion should be understood not only as an adaptive response to system pressure, but also as a modifiable contributor to low-value care. In the CathIRU study, the definition of an indicated PIVC explicitly accounted for anticipatory use in predefined high-risk situations; PIVCs inserted in patients at risk of deterioration were therefore considered appropriate, even if ultimately unused. Despite this, a substantial proportion of PIVCs remained classified as non-indicated, suggesting that anticipatory practices frequently extend beyond clinically justified scenarios.^1^ Available evidence further challenges the assumption that early PIVC insertion is necessary to avoid delays in care. Interventional studies have shown that fewer PIVCs can be inserted while increasing the proportion that are actually used, without reducing clinically appropriate indications. Recent prospective data indicate that more than half of PIVCs inserted in EDs are unnecessary or unused, while clinical deterioration requiring escalation of care remains rare (<1%).^2^^,^^3^ In addition, catheters prescribed “just in case” are significantly more likely to remain unused, and physicians can accurately predict non-use in most cases.^4^ Together, these findings suggest that anticipatory catheterisation reflects not only adaptation to system pressure, but also constitutes a driver of low-value care. Importantly, anticipatory PIVC insertion is not a neutral strategy from a workload perspective. PIVC insertion is more time-consuming than simple venepuncture and entails ongoing maintenance throughout the ED stay. When ultimately unused, these downstream requirements may paradoxically increase workload rather than alleviate it.

We also agree that expanding non-invasive therapeutic options, such as intranasal analgesia, represents a valuable and promising strategy.^5^ However, its applicability remains limited to a relatively narrow range of medications, primarily analgesics, despite pain being a leading reason for ED presentation. Furthermore, differences in bioavailability require adapted dosing strategies and access to multiple drug concentrations, which may increase the risk of administration errors in high-pressure environments. Implementation therefore requires appropriate training and careful integration into clinical workflows.

Ultimately, reducing non-indicated PIVCs will require aligning system design, therapeutic alternatives, and decision-making processes to support timely care while avoiding low-value interventions. Future strategies should focus on integrating decision-support tools at triage, expanding non-invasive pathways, and promoting deferred cannulation approaches in low-risk patients. While anticipatory PIVC insertion may represent an adaptive response to system constraints, adaptation should not be mistaken for an effective or sustainable solution to system-level challenges.

## Contributors

BD and OM contributed equally to the writing of this piece.

## Declaration of interests

We declare no competing interests.
